# Controlling molecular transport in minimal emulsions

**DOI:** 10.1038/ncomms10392

**Published:** 2016-01-22

**Authors:** Philipp Gruner, Birte Riechers, Benoît Semin, Jiseok Lim, Abigail Johnston, Kathleen Short, Jean-Christophe Baret

**Affiliations:** 1 Max-Planck-Institute for Dynamics and Self-Organization, Droplets, Membranes and Interfaces, Am Fassberg 17, DE-37077 Goettingen, Germany; 2 CNRS, Univ. Bordeaux, CRPP, UPR 8641, 115 Avenue Schweitzer, 33600 Pessac, France; 3 Laboratoire de Physique Statistique, Ecole Normale Supérieure, UPMC Univ Paris 06, Université Paris Diderot, CNRS, 24 rue Lhomond, 75005 Paris, France; 4 School of Mechanical Engineering, Yeungnam University, Gyeongsan 712-749, Republic of Korea

## Abstract

Emulsions are metastable dispersions in which molecular transport is a major mechanism driving the system towards its state of minimal energy. Determining the underlying mechanisms of molecular transport between droplets is challenging due to the complexity of a typical emulsion system. Here we introduce the concept of ‘minimal emulsions', which are controlled emulsions produced using microfluidic tools, simplifying an emulsion down to its minimal set of relevant parameters. We use these minimal emulsions to unravel the fundamentals of transport of small organic molecules in water-in-fluorinated-oil emulsions, a system of great interest for biotechnological applications. Our results are of practical relevance to guarantee a sustainable compartmentalization of compounds in droplet microreactors and to design new strategies for the dynamic control of droplet compositions.

## Abstract (web summary)

Emulsion droplets have many biotechnological applications, such as parallelized single cell analysis. Here, Gruner *et al*. introduce the concept of the minimal emulsions in a microfluidic device that allows full control of molecular transport between emulsion droplets.

## Graphical abstract

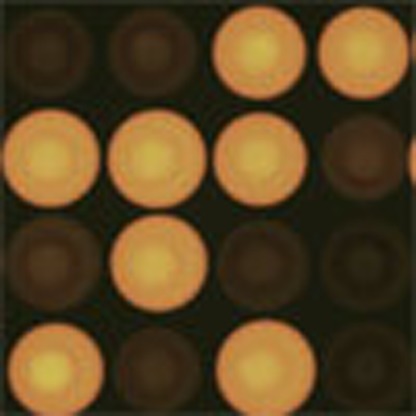

An emulsion is the dispersion of one fluid into another, stabilized by surfactant molecules^1^,^2^. Emulsions have a wide range of technological applications, including the use in food products, paints, cosmetics, chemical synthesis and drug delivery^3^. In recent years, droplet-based microfluidics has been proposed as a means for miniaturization and automatization of biochemical assays. The billions of microcompartments contained in an emulsion provide an environment ideal for the parallelization of assays^4^,^5^,^6^,^7^,^8^,^9^. This concept was shown to be very powerful for applications relying on high-throughput parallelized measurements such as drug screening^10^,^11^, biomarker analysis^12^,^13^,^14^, cell screening^15^,^16^,^17^ or directed evolution of enzymes^18^,^19^. These emulsions are unconventional in the sense that individual droplets have a unique and time-varying composition, depending on their initial loading and the biochemical processes occuring within them. From a physics perspective, emulsions are systems intrinsically out of thermodynamic equilibrium^1^,^2^. They are kinetically stabilized in a metastable state by the use of surfactant molecules. The role of the surfactant molecules is to increase the height of the energy barrier between the local energy minima of the system and its global minimum; this minimum is reached with a simple system where both phases are separated by an interface of minimal energy and in which the chemical potentials of all species is homogeneous. In addition to the classical ageing processes of flocculation, coalescence, gravitational separation and Ostwald ripening, solute transport drives these emulsions towards equilibrium. The heterogeneity in droplet composition leads to an increased number of local minima in the energy landscape and hence the relaxation to the global minimum can follow a complex path, resulting in complex dynamics.

We consider the kinetics of equilibration of concentration differences between droplets containing solutes which are poorly soluble in the continuous phase. We use fluorinated oils as the continous phase and aqueous droplets as the dispersed phase. This system is of particular interest for biochemical applications^20^,^21^. Although the solubility of organic molecules in fluorinated oils is normally very low^22^,^23^, surfactant molecules mediate their solubility, as they do for organic systems^24^, through their amphiphilic character^20^. Thus, there is a finite solubility of encapsulated compounds in the continuous phase, which can lead to cross-talk between droplets. In droplet-based microfluidic experiments, the droplets are designed to be independent microreactors, with the assumption that encapsulated compounds are not leaking into the continuous phase. This assumption is not valid in the presence of cross-talk between droplets^25^,^26^,^27^,^28^,^29^. Accessing quantitative information on mass transport in emulsions is experimentally challenging as the microenvironments of droplets must be precisely controlled to allow for quantitative analysis. Microfluidics is a powerful approach in the production of calibrated emulsions, providing access to a wide range of time scales and experimental conditions^21^,^30^,^31^,^32^. We have previously demonstrated how to measure chemical equilibration in three-dimensional systems^33^. Macromolecular additives, solubilized in a fraction of droplets, were shown to concentrate organic solutes in these droplets^33^. This result suggests that the local chemical equilibrium between phases is reached faster than the equilibration of concentrations in each phase by diffusion. Surfactants acting as an energy barrier to phase partioning at the time scale of the whole transport process is therefore excluded.

To further reduce the complexity of the emulsion system, we produce, order and immobilize droplets in a controlled fashion^7^,^34^. We introduce the concept of minimal emulsions made of an assembly of fixed monodisperse droplets, with controlled centre-to-centre distances. The microenvironment of each droplet is precisely controlled, to a level unreachable in bulk emulsification. As a result, we access fundamental information on the rate-determining step of transport. We demonstrate that the transport processes follow a universal law based on Fickian diffusion, described using simple thermodynamic arguments. We further use our understanding of the process to effectively control chemical transport between microreactors. We demonstrate the simple control and programming of chemicals in emulsions for targeted delivery into droplets at rates compatible with the typical time scales of biochemical assays. To the best of our knowledge, this result is the first demonstration of the full control of molecular transport between emulsion droplets. Our approach provides the tools to actively control the composition of droplets, for example in the continuous exchange of buffer or media conditions, without the need to individually manipulate droplets^35^.

## Results

### Minimal emulsions

**Figure 1:**
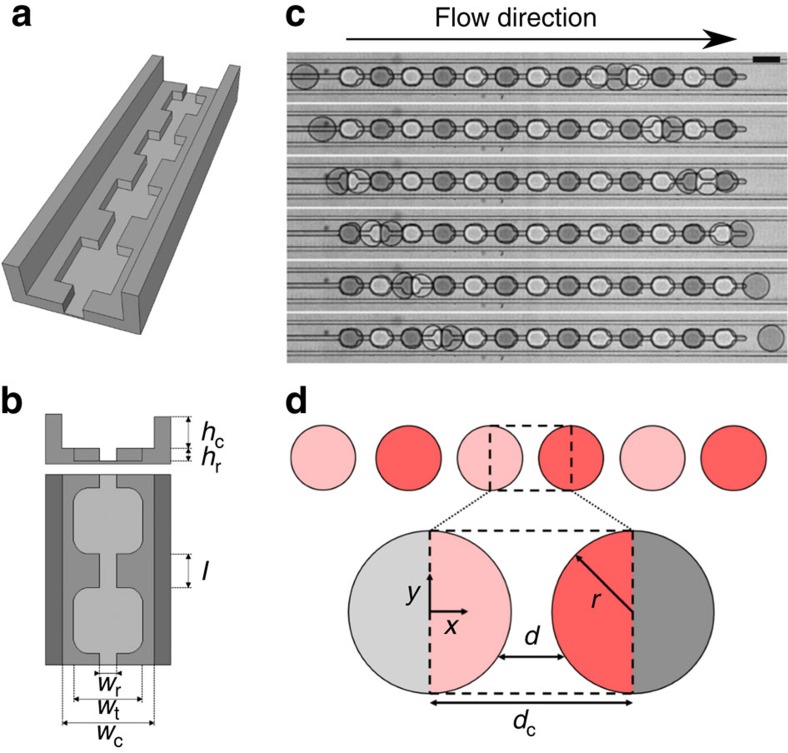
Microfluidics for the control of minimal emulsions. (**a**) Sketch of the trapping channel based on rails and anchors^34^,^42^,^43^. (**b**) The geometry of the channels determines the spacing distance between droplets (*h*_c_=20 μm, *h*_r_=15 μm, *w*_r_=20 μm, *w*_t_=80 μm, *w*_c_=100 μm, *l* varies between 36 and 6 μm). (**c**) Time sequence (100 ms interval) of droplets buffering each other from one local minimum in surface energy to the next (scale bar, 100 *μ*m). (**d**) Sketch of the membrane model used to describe the transport process in the array based on the transport through a single membrane (see Supplementary Fig. 2 and Supplementary Note 2).

Emulsions are prepared by dispersing one fluid into another. Shearing liquid–liquid mixtures in bulk leads to polydisperse emulsions while liquids co-flown in microfluidic channels form highly monodisperse emulsion droplets^36^. The monodispersity—crucial for many biochemical applications—is a key feature for exploring emulsion science^21^,^26^,^27^,^31^,^37^. We use these microfluidic techniques to control the order and spacing of the emulsion droplets reducing the number of degrees of freedom. In these ‘minimal emulsions', parameters such as the volume fraction of the dispersed phase are precisely defined. We prepare one-dimensional arrays of droplets with alternating composition in three steps. First, we produce a train of monodisperse droplets with alternating composition, then the train of droplet is actuated on-demand towards the storage and analysis zone, and finally the droplets are immobilized by halting the flow of the continuous phase. Alternating droplets are produced with two opposing T-junctions (Supplementary Fig. 1 and Supplementary Note 1). When one of the aqueous fluids is advancing into the nozzle, the other stream is blocked resulting in a reliable production of droplets with alternating properties^38^,^39^,^40^,^41^. To implement a function for on-demand switching of droplets, we design a pressure controlled hydrodynamic switch. The fluid flow is divided at a Y-junction into two microfluidic channels of different width. In the absence of any additional applied pressure, droplets preferentially flow towards the wider microfluidic channel owing to a lower hydrodynamic resistance^7^. Upon increase of the pressure level in the wider microfluidic channel, the pressure balance is inverted and droplets are directed towards the narrower channel (Supplementary Fig. 1). For the immobilization of droplets we use anchors and rails for guiding and storing droplets^34^,^42^,^43^ (Fig. 1a,b) and create regular arrays of droplets with controllable centre-to-centre distances *d*_c_ (Fig. 1c,d). Our system is thus reduced to the equivalent of a single membrane with periodic boundary conditions, given that all membranes between the droplets are equivalent and that edge effects are negligible^33^.

**Figure 2:**
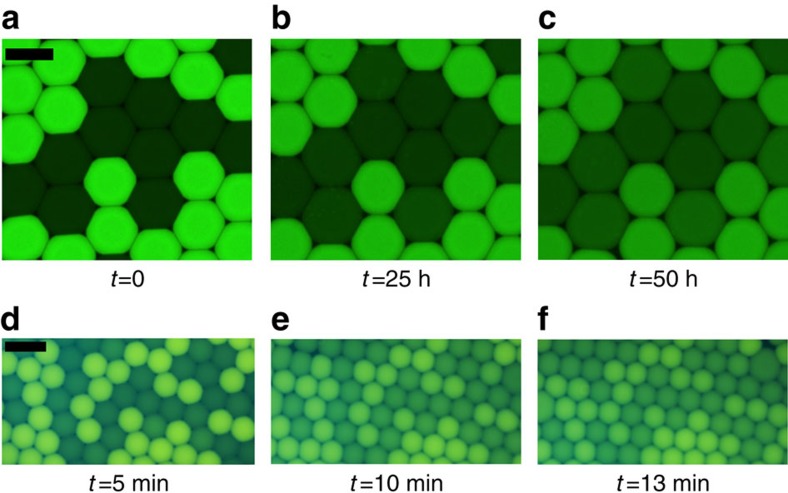
Molecular transport for two fluorophores. Fluorescein (**a**–**c**) is exchanged at a time scale of order several days in a water-in-fluorinated oil emulsion stabilized by a PFPE–PEG–PFPE surfactant (in HFE-7500). At production, the bright droplets contain Fluorescein at 100 μM in PBS water and the dark droplet PBS only. In contrast, Rhodamine 6G (**d**–**f**) is exchanged over minutes (scale bar, 100 μm). At production, the bright droplets contain Rhodamine 6G at 100 μM in millipore water and the dark droplet millipore water only. Resorufin is an intermediate case with a time scale of exchange of the order of 1 h (ref. 33).

**Figure 3:**
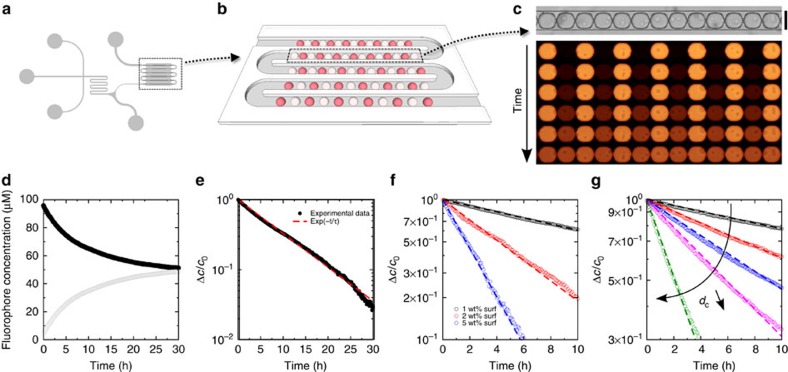
Fabrication and control of minimal emulsions. (**a**) Sketch of the device for droplet production and storage as alternating row (see Supplementary Fig. 1 and Supplementary Note 1). (**b**) On one single chip, several packing distance are tested under the same condition. (**c**) White-light micrograph of an immobilized row of droplets (scale bar, 100 μm) and time evolution of one row of droplets (0, 1.5, 3, 6, 12 and 24 h after immobilization, for a surfactant concentration *C*=1 wt% and a distance *d*_c_=4 μm.) in fluorescence. (**d**) Relaxation to equilibrium of the average fluorophore concentrations of initially filled (filled circles) and empty droplets (empty circles) (**e**) The concentration difference between the two droplet populations decays exponentially. (**f**) Variation of the kinetics as a function of surfactant (surf) concentration (for *d*_c_=15 μm). (**g**) Relaxation dynamics of five one-dimensional droplet microarrays for edge-to-edge distance *d*_c_−2*r* between the droplets of 30 μm (black), 15 μm (red), 10 μm (blue), 4 μm (pink) and 1 μm (olive) (for *C*=1 wt%).

We use fluorophores as model molecules. We will focus on three molecules, fluorescein, resorufin and rhodamine 6G which are all exchanged but exhibit time scales of exchange well separated, from minutes for rhodamine 6G to days for fluorescein (Fig. 2c,d). Resorufin is known to display exchange with a time scale of hours and will be used as a model dye for convenience^33^. Two droplet populations of identical size are produced, where fluorophores are only present in one population (resorufin sodium salt, 100 μM). The surfactant is a perfluoropolyether-polyethylenoxide block copolymer (PFPE–PEG–PFPE) stabilizing our emulsions against coalescence. The intensity of the emitted fluorescent light is proportional to the concentration of the fluorophore in the relevant concentration range between 0.1 and 100 μM. Hence, the concentration of resorufin sodium salt in individual droplets is determined by the fluorescence intensity. Droplets are stored in our arrays and fluorescence images are recorded every 5 min until the fluorophore concentrations between the two populations are equilibrated (Fig. 3a–c). The dynamics of transport of fluorophores from the initially ‘filled' towards the initially ‘empty' droplets are measured by analysing time sequences of fluorescence images.

**Figure 4:**
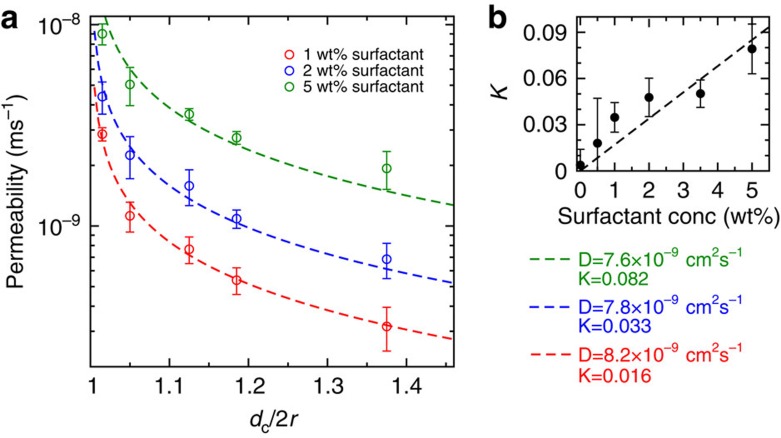
Oil Permeability *P*. (**a**) The permeability is a function of the centre-to-centre distance between the droplets *d*_c_ normalized by the droplet diameter 2*r*. Experimental data are fitted using equation (1) (see Supplementary Fig. 2 and Supplementary Note 2) to obtain the diffusion coefficient of the dye in the oil-surfactant mixture using the values of *K* determined independently (**b**). We obtain *D*=7.6 × 10^−9^ cm^2^ s^−1^ (5% surfactant), *D*=7.8 × 10^−9^ cm^2^ s^−1^ (2% surfactant), *D*=8.2 × 10^−9^ cm^2^ s^−1^ (1% surfactant). The value indicates that transport is mediated by surfactant aggregates affect the partitioning coefficient of the organic molecule between both the phases (error bars are standard deviation over three experiments); conc, concentration.

The concentration difference between the two droplet populations decays exponentially (Fig. 3d,e). The sum of intensity over all droplets stays constant during the experiment (Fig. 3d), indicating that photobleaching is negligible and that the concentration of the dye in the continuous phase is negligible compared with the concentration in the droplet. This latter point is expected from the low solubility of organic molecules in fluorinated oils. This result experimentally confirms that the alternating one-dimensional array is an eigenmode of the discrete diffusion equation considering that the transport occurs as a diffusive process through membranes arranged at the node of a one-dimensional lattice^33^. The time scale of the exchange in the array is therefore directly linked to the permeability of one single membrane. In addition, we recover that the time scale of the transport is inversely proportional to the concentration of surfactant (Fig. 3f; ref. 33). Next, we perform measurements of the relaxation to equilibrium for various droplet spacing, from ≈1 μm (touching droplets) to 30 μm (Fig. 3g). In all cases, the relaxation is exponential and the time scale 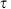 decreases as the spacing increases. Quantitatively, the time scale of exchange is related to the membrane permeability *P* using 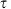
=*V*/*SP*^33^, where *V* is the droplet volume and *S* its surface area. Figure 4 shows the permeability as a function of the dimensionless spacing between the droplets *δ*=*d*_c_/2*r* (where *d*_c_ is the centre-to-centre distance between the droplets of radius *r*), for various concentrations of surfactant. For a value *δ*=1, undeformed spherical droplets would touch leading to infinite permeabilities in the diffusion-limited model. We observe experimentally a strong increase of the permeability as the droplets approach towards contact (Fig. 4), but the limit *δ*=1 is not reached as the droplet deform.

### Permeation model

In principle, the process of molecular exchange is predicted by thermodynamics: the chemical potential of the solute must equilibrate over the whole system through molecule transport. The process of molecular exchange involves four steps^44^: in the first step, solute molecules diffuse towards the water–oil interface; in the second step, the molecules partition between the water and the oil phase in the vicinity of the interface; third, the molecules diffuse through the oil phase towards a neighbouring droplet; and finally, as a fourth step, the molecules partition again, between the oil and the initially ‘empty' droplet until the equilibrium is reached. To date, the rate-determining step of molecular transport in water-in-oil emulsions has not been explicitly identified. However, recent numerical simulations have suggested that the transport of molecules across the phase boundary is the rate-limiting step^45^. In our experiments, the kinetics of the equilibration across the interface is constant and only the diffusive time scale in the oil varies. Qualitatively, since we observe a decay of the transport rate, the process must be diffusion limited. Quantitatively, we analyse the equilibration kinetics in our geometry for a purely diffusive process (that is, the first, second and fourth steps are significantly faster than the third). To understand the influence of droplet spacing on the time scale of transport, we use a permeation model (Supplementary Fig. 2 and Supplementary Note 2). For two reservoirs separated by an infinite membrane of thickness *l*, the permeability *P* of the membrane is given by *P*=*KD*/*l*, where *D* is the diffusion coefficient in the membrane and 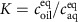 is the partitioning coefficient of the molecule between the membrane and the reservoirs. In our geometry, the membrane is not described by the single parameter *l* but includes two parameters, the spacing between the centres of the droplets *d*_c_ and their radius *r*. For simplicity, we approximate the trapped droplets as cylinders. From the geometry of the problem, we consider only the contribution of the gradient in the direction of the droplet–droplet centres expressed in a dimensionless form using *δ*=*d*_c_/2*r* (Supplementary Note 2):


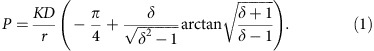


We compare the analytical expression equation 1 to our experimental results in Fig. 4. Qualitatively, the shape of the model curve fits the experimental data over the whole range of parameters tested. For quantitative comparison, we fit the experiments with a single fit parameter *D*, the diffusion coefficient of the dye in the oil. The value of *K* for the various surfactant concentrations (1, 2 and 5 wt%) is obtained independenty from the shake-flask method via spectroscopy (Fig. 4). The values of *K* are systematically smaller than 0.1, confirming the low solubility of resorufin in the oil phase. In addition, we found, to a first approximation, a linear variation of *K* with the surfactant concentration. Our results show that surfactant molecules mediate the solubility of organic molecules in fluorinated oils and play the key role for the transport of organic molecules in fluorinated emulsions. The values of the diffusion coefficient *D* obtained as a fitting parameter are in all cases *D*=7.9±0.3 × 10^−9^ cm^2^ s^−1^. Considering its size, the freely diffusing fluorophore is expected to diffuse with a diffusion coefficient on the order of 10^−5^ to 10^−6^ cm^2^ s^−1^. We therefore conclude that the diffusing object is much bigger than a single fluorophore molecule. With the Stokes–Einstein equation, we estimate an average diameter of ∼200 nm for the diffusing object. This size correlates with the size of surfactant assemblies obtained via dynamic light scattering (∼200 nm, See Supplementary Fig. 3 and Supplementary Note 3). We therefore conclude that assemblies of surfactant molecules, such as reverse micelles or vesicles, provide a nanoscopic environment for the solubilization of organic molecules in fluorinated oils and mediate the transport of small organic molecules. Our expression for the permeability shows a divergence close to droplet contact with a scaling *P*∼(*δ*−1)^−1/2^ (Supplementary Fig. 2 and Supplementary Note 2). Physically, we expect two mechanisms to limit the divergence. First, our hard sphere model will break down close to contact. Indeed, forcing the oil film between the droplets is made at the expense of the increase of pressure in the film. Droplets will ultimately deform when the capillary pressure *γ*/*r* is of the order of the film pressure leading to a non-zero spacing. Second, the partitioning between the oil and the water phases might become the limiting step. However, we will show that this transfer occurs at a much shorter time scale and is not the limiting step in most of the cases.

### Flow-induced targeted delivery

**Figure 5:**
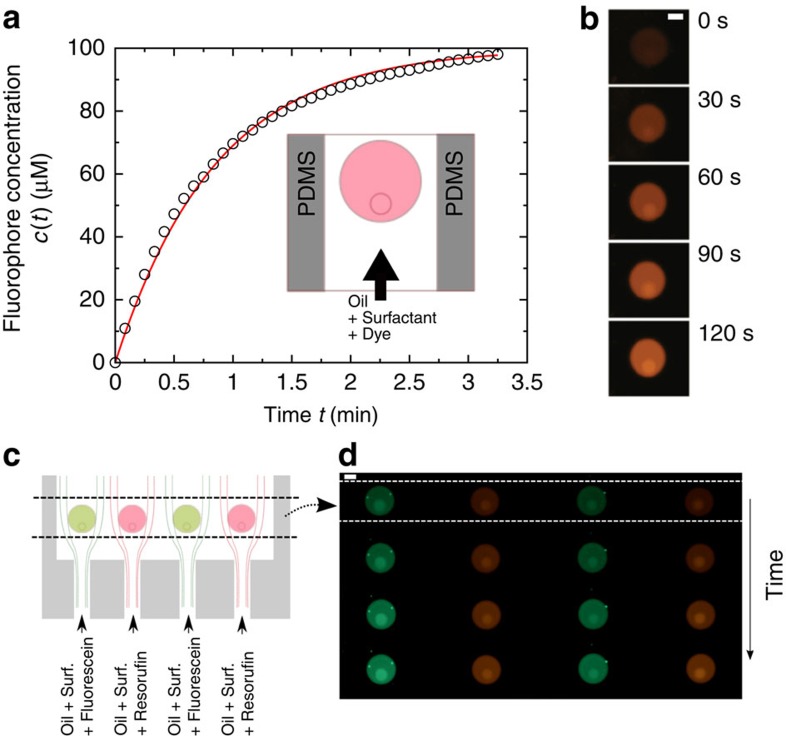
Flow-induced targeted delivery. (**a**) A droplet trapped in a microchannel is exposed to an oil doped with fluorescent dye (see inset). (**b**) The partitioning to the droplet leads to an increase of fluorescence (scale bar, 40 μm). The rate of molecule uptake by the droplet is given by the delivery rate from the oil (*Q*_oil_=0.2 μl min^−1^). The principle for a controlled uptake of multiple dyes is shown in **c**. (**c**) Droplets of 100 μm in diameter are trapped in a microfluidic chamber. We selectively inject fluorescent dyes (fluorescein and resorufin) from the oil and surfactant mixture into the droplet. The dye injected depends on the position of the droplet in the chamber. (**d**) Experimental realization: fluorescent micrograph of the droplets showing the dye uptake occuring at a time scale of the order of 1 min. Here *Q*_oil_=0.2 μl min^−1^.

To measure the rate of transport accross the oil–water interface, we use a second microfluidic approach. By doping the fluorous continuous phase with resorufin, making use of the surfactant-induced solubility, we induce the uptake of resorufin by a droplet trapped in a microchannel (Fig. 5). The time scale of uptake is measured as 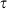
∼45 s, and is several orders of magnitude smaller than the rates measured in emulsions. Interestingly, it is directly given by the flux of dye carried by the oil. This result indicates that even for short time scales, there is no significant effect of the partitioning kinetics to slow down the transport across the interface. From this time scale, we can construct a permeability 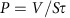
∼10^−6^ m s^−1^ (for a droplet size of 50 μm in diameter). This permeability is at least two orders of magnitude larger than the permeabilities obtained in Fig. 4, finally showing that the transport in emulsions is not influenced by the kinetics of partitioning. The transport process is entirely limited by the fluorophore transfer through the continuous phase, from spaced droplets to almost touching droplets, in flow or in quiescent conditions. There is no significant influence of a kinetic barrier to the partitioning at the interface, in contradiction to recent theoretical models^45^. We further use our approach to demonstrate that droplets trapped in a wide channel are selectively fed by chemicals, using a proper design of microchannels (Fig. 5c,d). In this case, solutions of oil and surfactant doped with resorufin or fluorescein are injected towards immobilized droplets. The laminar flow provides a control of the delivery towards specific droplets. Here, we obtain a direct control of the droplet composition through the control of the continuous phase.

### Emulsion-based targeted delivery

As the partitioning coefficient between the dispersed and the continous phase is the important control parameter for mass transport in emulsions, acting on partitioning coefficients is a key concept to control transport rates. We have previously shown that BSA (bovine serum albumin) added to the aqueous phase slows down the rate of transport through partitioning^26^,^33^. In other systems, sugar additives have been used to slow down the transport^29^. The solubility of organic molecules in an aqueous medium is also altered by the addition of salt^46^, commonly known as the ‘salting out' effect. By increasing the salt concentration, more water molecules are involved in solvation, decreasing the number of water molecules available to hydrate the fluorophores. As a result, the solubility in the aqueous phase decreases with increasing salt concentration.

**Figure 6:**
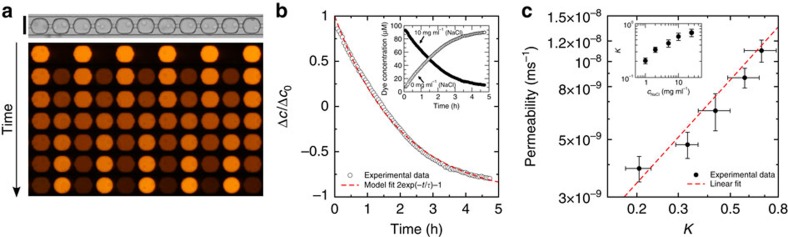
Emulsion-based targeted delivery. (**a**) White-light picture of an immobilized row of droplets and time sequence of fluorescence signals (0, 0.5, 1, 1.5, 2, 2.5, 3 h after immobilization, scale bar, 100 μm). (**b**) Relaxation of the concentration difference towards the opposite composition. A complete inversion of the concentration is obtained according to an exponential relaxation for droplets initially containing 100 μM resorufin sodium salt and 10 mg ml^−1^ sodium chloride (filled circles, see inset) and millipore water droplets (empty circles, see inset). (**c**) Dependency of permeability *P* on the partitioning coefficient *K*: the transport rate is driven by the largest partitioning coefficient in the system. Inset: dependency of partitioning coefficient *K* on the salt concentration in the aqueous phase *c*_NaCl_ (error bars are standard deviation over three experiments).

We use this principle to design a scenario where we obtain the transfer of fluorophores from one droplet population to the other (Fig. 6a,b). While one population initially contains fluorophores and sodium chloride (1–25 mg), the other population does not contain any additives. Consequently, the partition coefficient of the fluorohpores is different between the two droplet populations (Fig. 6c). We observe that the fluorophores are enriched in the droplets free of sodium chloride. We obtain an exponential relaxation for the concentration difference from the initial state to the reversed state (Fig. 6a,b). An equal concentration is obtained transiently in the course of the exchange (at times of the order of 1 h in this case). At a later stage, the final ratio of concentrations in both types of droplets is given by the ratio of the partitioning coefficients with and without salt. Quantitatively, we again correlate the permeability of the oil with the partitioning coefficient (Fig. 6c). Finally, it should be noted that the final state of the system is not given by two populations of droplets of the same size. At longer times, the transport of the dispersed phase itself becomes significant to balance the osmotic pressure difference related to the imbalance of salt concentration^30^,^47^,^48^. Therefore, our method provides means to circulate along various paths of the relaxation to the equilibrium of our dispersion.

**Figure 7:**
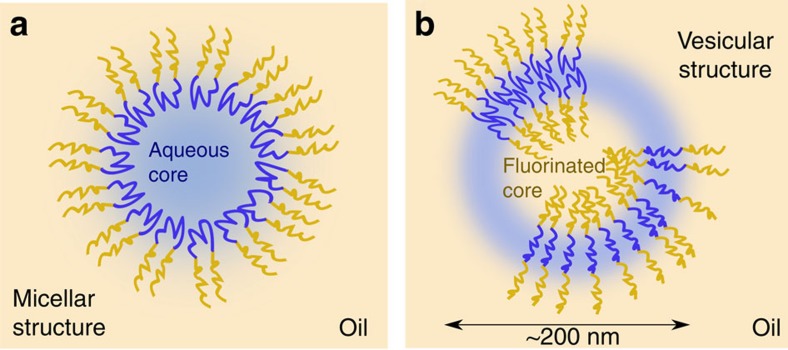
The possible structures of the surfactant supramolecular assemblies. (**a**) The swolen micelle structure with an aqueous core, (**b**) the vesicle structure where the surfactant is either extended or forms bilayers. The vesicle structure is compatible with our experimental results. With the micelle structure, the exchange rate of water should depend on surfactant concentration and all dyes should have the same exchange rates (see Supplementary Fig. 4 and Supplementary Note 4).

It is interesting to compare the transport of organic molecules to the transport of the dispersed phase itself. Ostwald rippening is observed in fluorocarbon oils but the kinetics of transport is independent of the surfactant concentration (Supplementary Fig. 4 and Supplementary Note 4). As a consequence, there is no significant increase of water solubility with increasing surfactant concentration. Therefore, the structure of the supramolecular assemblies is not compatible with water-filled micelles, but is rather compatible with an oil-in-oil vesicle-type assembly as observed for organic block copolymers^49^ (Fig. 7). This structure is also compatible with the experimental observation that various dyes are exchanged with different time scales^26^,^33^.

### Surfactant-based chemical extraction

**Figure 8:**
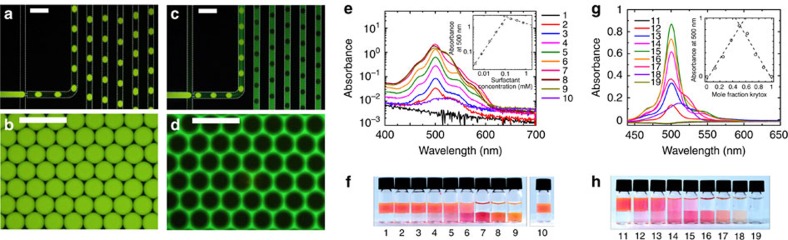
Surfactant-mediated extraction of solutes. (**a**–**d**) Fluorescence images of microfluidic droplet production and resulting emulsions in the presence of 0.5% PFPE–PEG–PFPE surfactant and additionally low (**a**,**b**<1%) or high (**c**,**d**=30%) mass fractions of carboxylic acid fluorosurfactants (scale bar, 300 μm). The aqueous and fluorous phase flow rate are 1 and 4 μl min^−1^, respectively. (**e**–**h**) Macroscopic partitioning experiments. (**e**) Absorption spectra of the fluorous phase after equilibration shown for various concentrations of the additive. The inset shows the absorption at 500 nm. (**f**) Corresponding images of the partitioning experiments. Aqueous solutions of rhodamine 6G (initial concentration: 100 μM) are exposed to the fluorous phase with additive concentrations from 1 to 9 as: 0, 3.75, 7.51, 18.8, 37.5, 93.8, 188, 375 and 751 μM. Solution 10 contains only the PFPE–PEG–PFPE surfactant at a concentration of 877 μM (0.5 wt%). (**g**) Absorption spectra of the fluorous phase for various mole fractions of fluorophore and surfactant. Inset: absorbance at 500 nm as a function of the mole fraction of the additive (Job plot). (**h**) Images of the corresponding partitioning experiments. In samples 11–19, the fraction of the additive increases as: 0, 0.125, 0.25, 0.375, 0.5, 0.625, 0.75, 0.875, 1.0.

To gain additional insights into the surfactant-mediated solubility, we performed a final set of experiments. Here, we address the opposite case, namely the extraction of compounds from droplets. The principle derived from our previous experiment is to increase the partitioning towards the oil phase to extract the molecules. Emulsions were formed with 0.5 wt% of PFPE–PEG–PFPE surfactant comprising an additive, a carboxylic acid surfactant (Krytox-FSH) at low (<1%) and high (30%) mass fractions. To visualize the extraction of compounds from droplets, the organic fluorophore rhodamine 6G was added to the aqueous phase. The fluorophore was chosen for two major reasons: first, it is fluorescent in both phases, and second, it is a water soluble molecule, that does not partition into the pure fluorous phase. Interestingly, we observe that the efficiency of the fluorophore extraction strongly depends on the surfactant type. In the presence of the sole PFPE–PEG–PFPE surfactant (0.5 wt%), the fluorophore molecules are retained in the droplet (Fig. 8a,b). In contrast, the addition of the carboxylic acid surfactant to the PFPE–PEG–PFPE surfactant with a surfactant fraction of 30% results in an immediated and complete extraction of the fluorophores into the continuous phase (Fig. 8c,d). In microfluidics, the process of extraction is completed within less than 1 s (Fig. 8c,d). This experiment shows a direct control on the process of extraction by additives solubilized in the fluorous phase and confirms that the time scale for partitioning is several orders of magnitude shorter than the time scale of the diffusive process.

### Unravelling relevant interactions

In addition, the use of rhodamine 6G sheds light onto the mechanism of partitioning. To quantitatively analyse the extraction of the fluorophores into the fluorous phase, aqueous solutions of rhodamine 6G (100 μM) are exposed to a fluorous phase (HFE7500) containing various concentrations of the carboxylic acid surfactant (Fig. 8e,f). In the absence of surfactant, no significant extraction of molecules is observed. With increasing concentration of the carboxylic acid surfactant, the amount of organic molecules being extracted into the fluorous phase is increasing up to full extraction (>95%). The absorbance in the fluorous phase is proportional to the concentration of surfactant up to about 100 μM. Above this 1:1 molar ratio, no further increase in absorbance, but a bathochromic shift is observed. The acid acts as a molecular receptor for rhodamine 6G resulting in the efficient extraction of the organic solutes into the fluorous phase.

To validate the stoichiometry of the complex formed in the continuous phase from the two solute species present in the system, a continuous variations experiment is conducted (Fig. 8g,h). The total molar concentration of the acidic surfactant and the fluorophore is constant, but their mole fractions are varied. The maximum of absorption in the fluorous phase is found at a 1:1 molar ratio indicating that a complex with the corresponding 1:1 stoichiometry is formed.

Specific noncovalent interactions are known to increase the solubility of organic molecules in fluorous liquids^50^ through hydrogen bonding or ion pairing. Fluorosurfactants with a carboxylic acid head group form strong hydrogen bonds with organic molecules^51^,^52^. Fluorous carboxylic acids act as molecular receptor for organic molecules significantly increasing their solubility in fluorous liquids^53^. Nitrogen hydrogen bond are also efficient acceptors^51^, as shown for pyridines^51^,^52^. In summary, noncovalent interactions significantly improve the extraction of organic molecules into a fluorous phase and the efficiency is strongly dependent on the compatibility of substrate and receptor.

This result obtained with a seemingly different system compared with the PFPE–PEG–PFPE molecule used above is important to explain the interactions within the supramolecular assemblies of the block copolymer. The amide group binding the fluorinated chains to the PEG group is a good candidate to be at the origin of the interactions between the surfactant assemblies and the solutes. We want to point out that although hydrogen bonding is a good candidate to explain the dye–surfactant interactions, obtaining a direct a priori correlation between structure and exchange rate remains challenging.

With respect to applications of droplet-based microfluidics, it becomes clear that impurities of carboxylic acids can have a tremendous effect on the performance of the compartmentalization system. Hence, characterizing the amount of carboxylic acid remaining after synthesis is crucial. Various methods such as NMR and IR measurements may be applied. However, these methods may not be sufficiently sensitive to trace impurities in a concentration range relevant for typical assays applied for biotechnological purposes (<100 μM). Phase partitioning of a fluorescent indicator, such as rhodamine 6G, from a more protic solvent may be a sensitive method for the determination of the concentration of the residual carboxylic acid. The analysis of several surfactant batches by our partitioning method shows how the level of traces of side products in surfactant samples is determined (Supplementary Fig. 5 and Supplementary Notes 5 and 6). Finally, this chemical view of the interactions provides guidelines to design surfactants to guarantee efficient encapsulation or in contrast favour selective extraction of compounds from droplets.

## Discussion

Our experiments show that microfluidics provides tools to efficiently manipulate droplets to prepare, order and store them in a controlled manner; emulsions with a precisely defined microstructure are obtained for quantitative studies of physicochemical processes at the microscopic level. We show that the fluorophore transport in fluorinated emulsions, used as a model for organic molecule transport, is, in all our experiments, limited by the diffusive transport through the continuous phase. The dependence of the transport process on the droplet spacing is fully consistent with an analytical model based on the proper description of the permeability of the oil membrane separating the droplets. Increasing the spacing between droplets is an efficient strategy in reducing the exchange of material between droplets. In combination with a decrease of concentration of surfactant, we have shown a decrease in the rate of transport by a factor of about 30. In a bulk emulsion, the equivalent strategy would be to increase the continuous phase volume fraction which is technically challenging. The values of the diffusion coefficient of the fluorophores in the continuous phase obtained experimentally show that the transport of fluorophores is mediated by large assemblies of surfactant molecules. Simple additives, such as sodium chloride, BSA^33^ or sugars^29^, not only affect the rate of transport but also the distribution of organic molecules among the droplets. We demonstrate how to use this concept for the targeted delivery of compounds, a potential new mechanism for actively feeding droplets from external sources. In practice, special care should be taken when changing buffer conditions in biochemical applications or using additives such as encoding fluorophores which might affect interactions and partitioning. In contrast, understanding and controlling this process is essential to deliver molecules from one droplet to the next and might provide new tools for the chemical control of the content of emulsion droplets. Besides straightforward applications in droplet-based microfluidic systems, we believe that our approach will be applicable to emulsion-based synthesis where transport of reagents between compartments is crucial. Our system might also provide additional insights to understand how organic molecules can be concentrated in a population of microcompartments, a question relevant for compartmentalization through phase separation in cells^54^, for prebiotic chemical systems^55^,^56^ and for the design of minimal functional micro-compartments^55^,^57^,^58^.

## Methods

### Chemicals

Resorufin sodium salt, fluorescein and rhodamine 6G (Sigma-Aldrich) solutions were prepared by dissolution in millipore water or NaCl solutions. Droplets were produced in fluorinated oil (HFE-7500, 3M) and stabilized against coalescence by a perfluoropolyether–polyethyleneglycol block-copolymer surfactant (PFPE–PEG–PFPE, Critical Micellar Concentration ∼0.03% (weight fraction)^33^). The surfactant was a kind gift from Dr E. Mayot, prepared from the carboxylic acid Krytox (157-FSH, Dupont) and polyethyleneoxide (Sigma-Aldrich), adapting the synthesis scheme described by Holtze *et al*.^59^ and Scanga *et al*.^33^,^60^ The infra-red spectra of the surfactants used here, a detailed protocol for surfactant synthesis and the analysis of the rhodamine 6G partitioning towards the oil phase are provided in Supplementary Fig. 5 and Supplementary Notes 5 and 6. After mixing the surfactant in the fluorinated oils, the solutions are stable and stored in closed glass flasks and handled at room temperature.

### Microfluidic device fabrication

Chips were made of Norland Optical Adhesive 81 (NOA81). Briefly, a positive SU-8 mould is made by two-layer photolithography to fabricate a negative PDMS stamp by replica moulding. A drop of NOA81 is deposited on the patterned PDMS substrate. For curing, the liquid film is covered with a blank PDMS substrate and exposed to ultraviolet light (4 min, Polylux PT (Dreve)). For fluidic connection, holes are punched into the crosslinked NOA81 layer (Harris Uni-Core Punch, 0.75 mm). Subsequently, the layer is stuck to a glass substrate, which was previously coated with NOA81 glue (500 r.p.m.) and then crosslinked (4 min, Polylux PT (Dreve)), improving bonding. Ports (Upchurch) are glued on top of the punched holes. A second layer of NOA81, made in the same way, is put in contact with the first layer to provide overlying microfluidic chambers that can be filled with water during the experiment. The completed chip is exposed to ultraviolet light for 40 min (Polylux PT (Dreve)). Aquapel (PPG Industries) was used to hydrophobize the channels: the aquapel solution is used as received and simply injected from a gastight syringe into the channels. The channels are then dried with nitrogen.

### Device operation

A pressure-driven pump (Fluigent, MFCS-8C) was used to control the flow in the microfluidic device. For droplet production, the two aqueous solutions were co-flown with the fluorinated oil containing a distinct concentration of surfactant (1, 2 or 5 wt%). The pressure levels of the aqueous phases were set to 280 mbar each, the oil phase was set to 260 mbar. For on-demand direction of droplets at the hydrodynamic switch, the pressure level in the wider microfluidic channel was controlled. Up to typically 110 mbar, all droplets flow towards the wider microfluidic channel. Above ≈180 mbar, the behaviour is inverted such that all droplets flow through the narrow microfluidic channel towards the experimental zone of the chip. They buffer each other from one to the next local minimum in surface energy (Supplementary Fig. 1 and Supplementary Note 1). Lowering the switch pressure to 50 mbar leads to an immediate stop of delivery of droplets towards the experimental zone. However, the droplets are still buffering each other such that no droplets will be immobilized in between the local minima of surface energy. We immobilize droplets in five independent one-dimensional microarrays, each with varying distances between the droplets (0, 3, 10, 15, 30 μm edge–edge distance). Each microarray consists of 13 to 15 droplets.

### Fluorescence measurement and data processing

Images were taken every 5 min with a digital camera (Canon, EOS D600). A light emitting diode (CoolLED pE-2, 550 nm) was used for the excitation of fluorophores. The recorded intensity in the red channel was found to be proportional with the dye concentration in the relevant range (0–100 μM). Images were analysed with homemade scripts using the standard toolbox in Matlab.

### Flow-induced target delivery

We used two independent dyes (fluorescein and resorufin). Solutions of fluorescein (alternatively resorufin) are prepared as followed: a mixture of oil and surfactant (at 1%(w/w) concentration) is prepared and equilibrated for several days with a fluorescein (alternatively resorufin) solution at 100 μM in a closed vessel. At the end of the incubation, the oil is recovered and loaded in a syringe. Both solutions are injected in independent microchannels, feeding the microfluidic chamber towards empty droplets (that is, droplets containing only buffer). The laminar flow profile of the oil ensures that single droplets are targeted. When the droplet buffer composition is similar to the composition of the solution used during the incubation, the equilibrium concentration of the fluorescein (respectively, resorufin) is the concentration used initially.

### Measurement of partitioning coefficients

The partition coefficients between the aqueous and the fluorinated phase are determined by the shake flask method. A total 500 μl of HFE-7500 with a distinct concentration of surfactant (0.5–5 wt%) and the same amount of millipore water containing 100 μM of resorufin sodium salt and in some cases sodium chloride (0–25 mg ml^−1^) are put into contact in a glass vial. Careful pipetting ensures that no emulsification is obtained. The mixture of the two liquids is incubated for at least 72 h. The partition coefficient is calculated by measuring the change in fluorescence intensity of the aqueous phase with a microplate reader (Spectra Max Paradigm, Molecular Devices).

## Additional information

**How to cite this article:** Gruner, P. *et al*. Controlling molecular transport in minimal emulsions. *Nat. Commun.* 7:10392 doi: 10.1038/ncomms10392 (2016).

## Supplementary Material

Supplementary Information Supplementary Figures 1-5, Supplementary Notes 1-6 and Supplementary References

Supplementary Movie 1 Hydrodynamic switch in the off state. The droplets are directed to the right channel (waste).

Supplementary Movie 2 Hydrodynamic switch in the on state. The droplets are directed to the left channel (towards the storage chamber).

Supplementary Movie 3 Filling of the storage chamber by buffering and release. Six experimental conditions of droplet spacing are tested in one single run.

Supplementary Movie 4 Equilibration of resorufin concentration in the array.
