# Supplementary material for: Controlling molecular transport in minimal emulsions
Source: Nat Commun. 2016 Jan 22;7:10392. doi: 10.1038/ncomms10392 (PMC4735829; doi:10.1038/ncomms10392)
Supplement: Supplementary Information — Supplementary Figures 1-5, Supplementary Notes 1-6 and Supplementary References [file ncomms10392-s1.pdf]

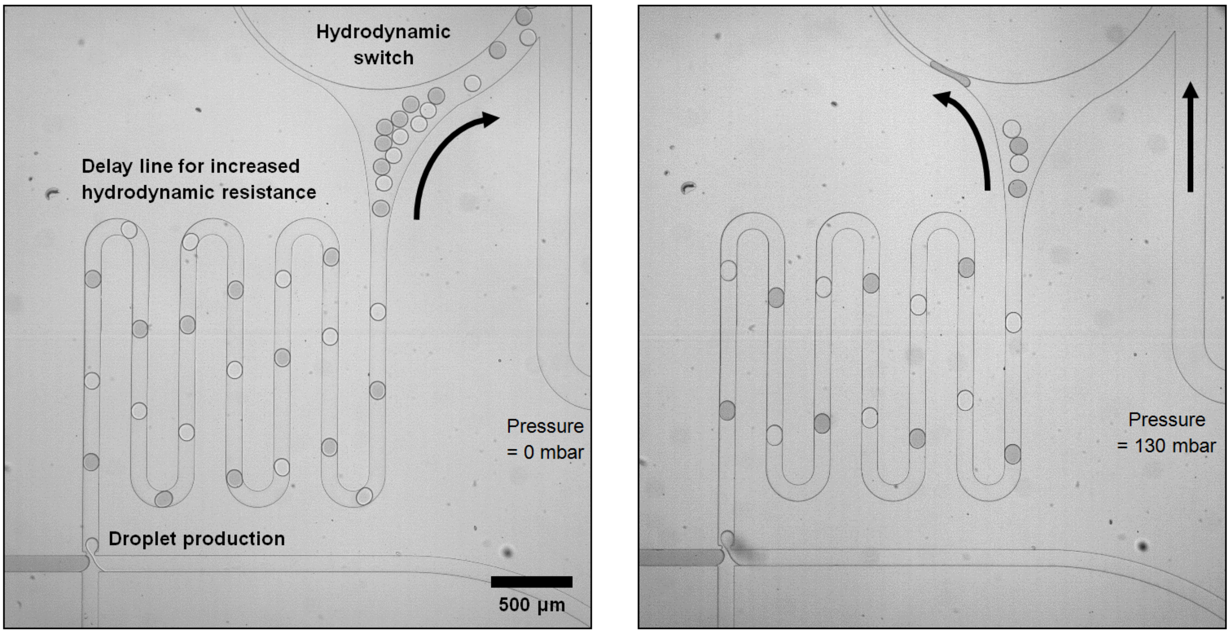

Supplementary Fig. 1: Working principle of the applied hydrodynamic switch. (Left) Droplets follow the path of lower hydrodynamic resistance in the absence of any additionally applied pressure in the wider channel at the switch. (Right) Droplets pass through the narrow channel at the switch and are directed to the experimental zone as a result of an increased pressure in the wider microfluidic channel (See Supporting Movies 1 and 2.)

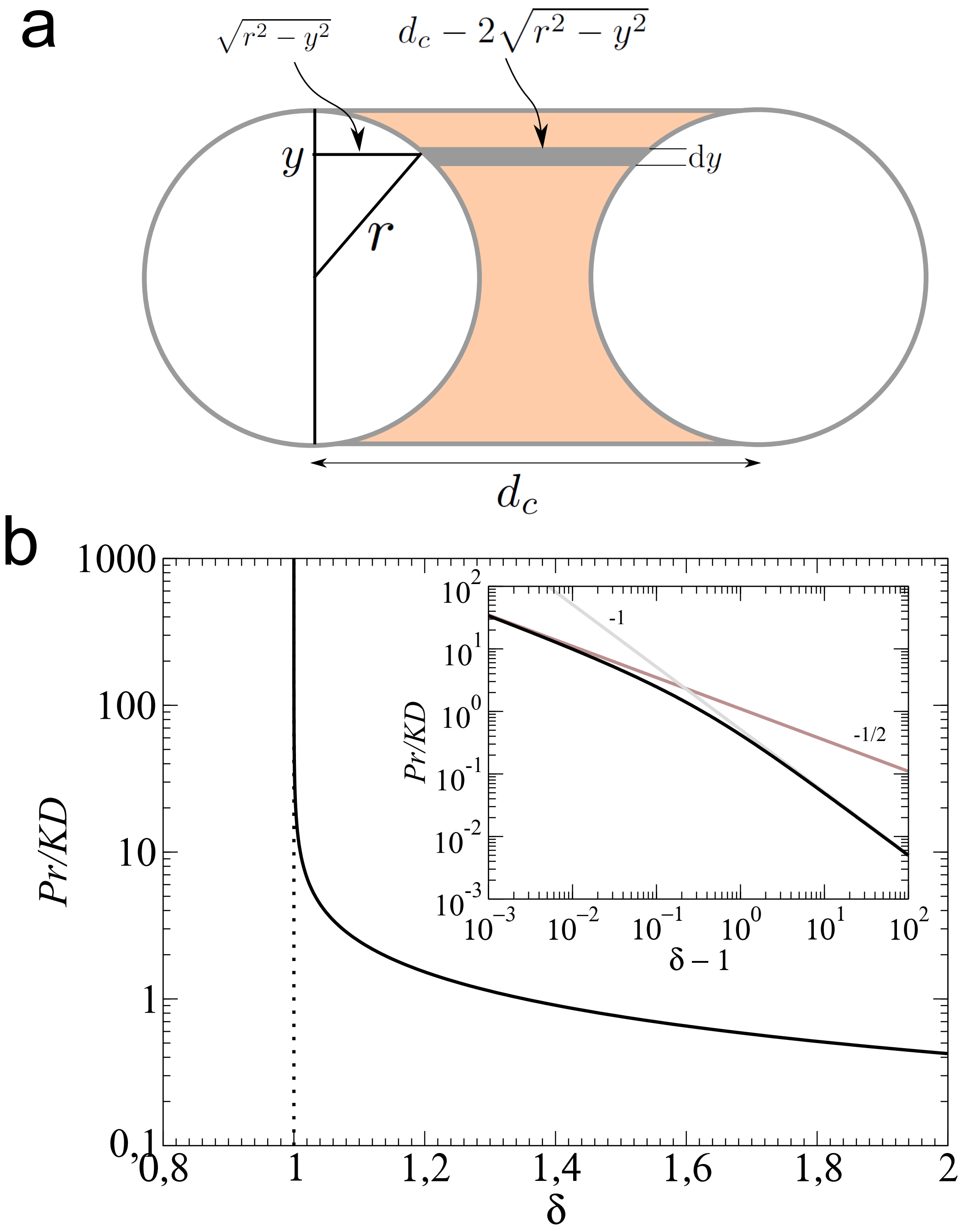

Supplementary Fig. 2: Permeability model. (a) Sketch of the membrane between two droplets. The droplets are considered as cylinders in the height. (b) Permeability of the oil membrane separating two droplets in a diffusion limited model. The permeability P diverges for touching droplets $\delta = 1$. The asympotic scaling shows a -1/2 power law exponent for touching droplets and a -1 exponent for sparse droplets ($\delta \to \infty$). The crossover is obtained for $\delta - 1 \sim 2/\pi^2$. The typical values for the permeability at the crossover is given by $P \sim \pi^2/4 \times KD/r$.

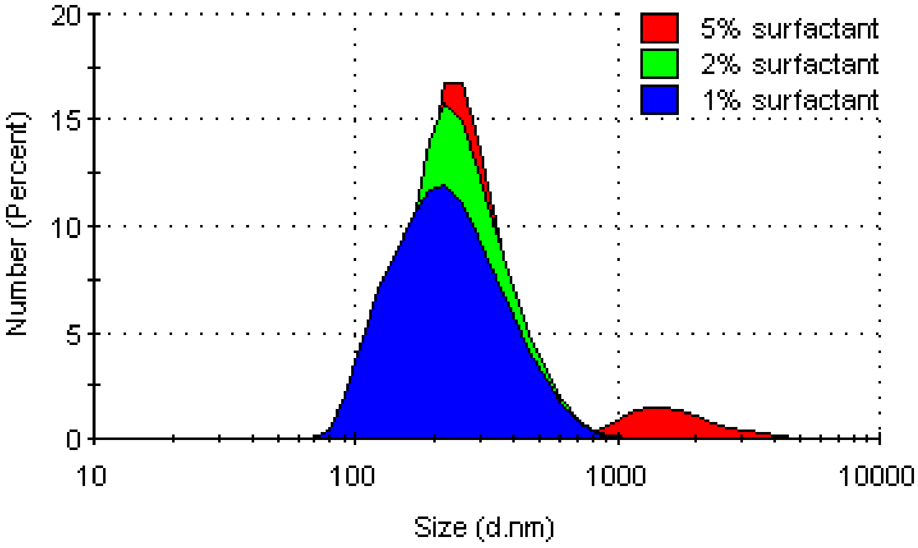

Supplementary Fig. 3: Size distribution of surfactant assemblies measured by dynamic light scattering shown for various surfactant concentrations.

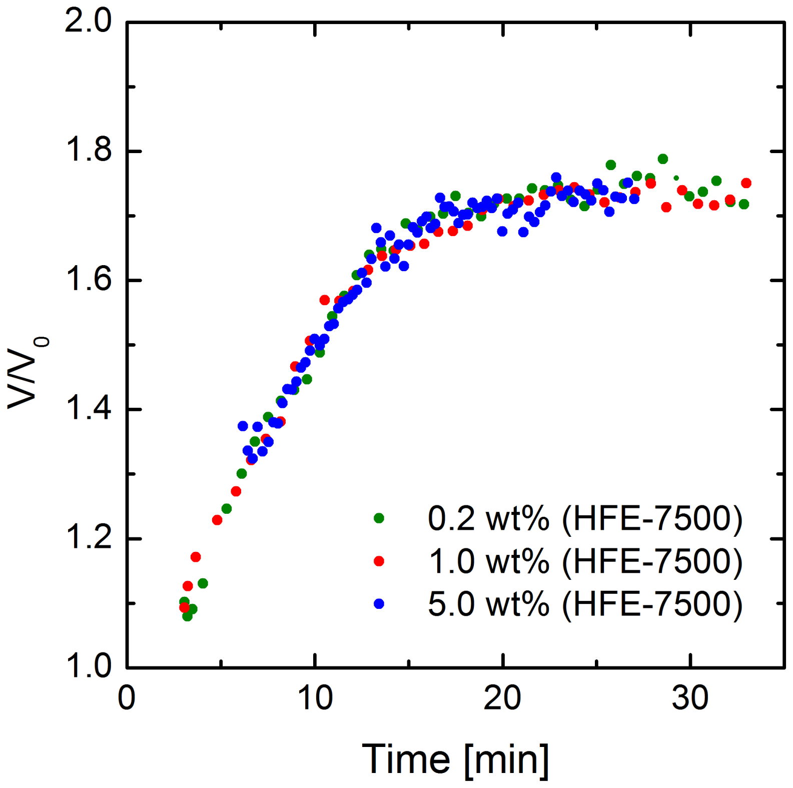

Supplementary Fig. 4: Water Transport: Volume increase of droplets containing sodium chloride by osmotically driven water transport, normalized by the initial droplet volume V_0. The water transport is independent on the surfactant concentration (0.2 wt%, 1.0 wt%, 5.0 wt%) in the fluorinated oil HFE-7500.

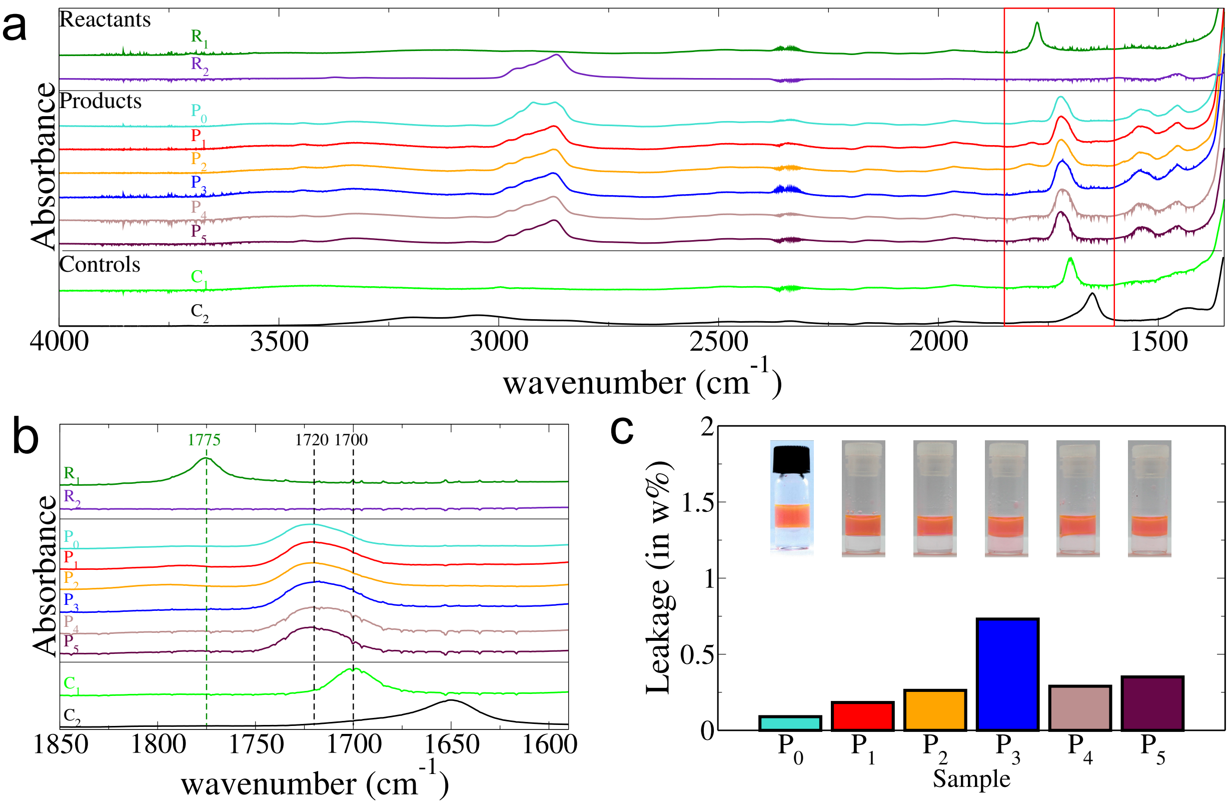

Supplementary Fig. 5: Analysis of the products of the reaction for the surfactant synthesis. (a) Infra-Red spectra of the reagents, products and control synthesis: the covalent binding of the Jeffamine and the PFPE is revealed by an IR band at 1720 cm^{-1}. The Jeffamine-PFPE salt corresponds to a band at 1700 cm^{-1} (see also Dejournette *et al.* [1]). The carboxylic acid band of the reactant is at 1775 cm^{-1} and is barely visible indicating traces of reactants in the final product. P_0 is the surfactant used in the main text. Details on the synthesis of P_{1-5} and the controls C_{1-2} are provided in the Supplementary Note 7. (b) Zoom of the carbonyl stretch bands. (c) Partitioning experiments used to determine the amount of residual Krytox. A rhodamine 6G solution is put in contact with a 0.5 wt% solution of the synthesised product dissolved in HFE-7500 (inset). The amount of Rhodamine 6G extracted into the oil is measured by visible spectroscopy to determine the amount of residual Krytox (Figure 7 (e,f) main text). The equivalent Krytox concentration in all synthesized products is less than 1%(w/w) confirming that it is at the level of traces.

Supplementary Note 1: Hydrodynamic switch

Droplets are produced in an alternating order in a microfluidic cross section by coflowing two aqueous streams with a stream of fluorinated oil (HFE7500) containing surfactant (1, 2 or 5 wt%) as shown in Supplementary Fig. 1. The following delay lines allow surfactant adsorption and increase the hydrodynamic resistance. This increase in resistance is essential as pressure variations at the switch would otherwise have a tremendous effect on droplet production. At the hydrodynamic switch droplets can either flow through a wide or a narrow microfluidic channel. In the absence of any additionally applied pressure, the droplets follow the path of lower hydrodynamic resistance through the wider microfluidic channel (Supplementary Fig. 1, left). When a sufficiently high pressure is applied in the wider microfluidic channel, droplets pass through the narrow channel (Supplementary Fig. 1, right) and are directed to the experimental zone. The typical pressure levels applied for droplet production were 280 mbar for each aqueous phase and 260 mbar for the fluorinated oil phase. When the pressure level in the wider microfluidic channel is increased to above 110 mbar, all droplets pass through the narrow microfluidic channel.

Supplementary Note 2: Derivation of the permeability

As a first approximation, we consider the spacing between droplets as a permeable membrane. In the third dimension, the droplets are squeezed and considered as cylinders (Supplementary Fig. 2). The permeability P is expressed as:

$$P = KD/l_{eq} \tag{1}$$

where l_{eq} is the equivalent thickness of the membrane between the droplets. We expect l_{eq} to be larger than the minimal distance between the droplets, namely $l_{eq} > d_c - 2r$. From geometrical arguments, l_{eq} is writen as

$$\begin{aligned} \frac{1}{l_{eq}} &= \frac{1}{2r}\int_{-r}^{+r} \mathrm{d}y \frac{1}{d_c - 2\sqrt{r^2 - y^2}} \\ &= \frac{1}{r}\int_{0}^{+r} \mathrm{d}y \frac{1}{d_c - 2\sqrt{r^2 - y^2}} \end{aligned}$$

using a symmetry argument. The typical scale of the problem being r, lateral dimensions are writen in a dimensionless form $\xi = y/r$. We introduce the dimensionless spacing $\delta = d_c/2r$, leading to :

$$\frac{2r}{l_{eq}} = \int_0^1 \frac{\mathrm{d}\xi}{\delta - \sqrt{1-\xi^2}}$$

To solve the integral, we make a first change of variable, introducing $\xi = \cos\theta$:

$$\mathrm{d}\xi = -\sin\theta \mathrm{d}\theta$$

$$\xi = 0 \Leftrightarrow \theta = \pi/2$$

$$\xi = 1 \Leftrightarrow \theta = 0$$

Therefore:

$$\frac{2r}{l_{eq}} = \int_{\pi/2}^{0} \frac{-\sin\theta}{\delta - \sqrt{\sin^2\theta}} \mathrm{d}\theta \tag{2}$$

Since $\theta \in [0, \pi/2]$, $\sqrt{\sin^2\theta} = \sin\theta$ and:

$$\begin{aligned}
\frac{2r}{l_{eq}} &= \int_0^{\pi/2} \frac{\sin\theta}{\delta - \sin\theta}\mathrm{d}\theta \\
&= \int_0^{\pi/2} \frac{\delta + \sin\theta - \delta}{\delta - \sin\theta}\mathrm{d}\theta \\
&= \int_0^{\pi/2} \frac{\delta}{\delta - \sin\theta}\mathrm{d}\theta - \int_0^{\pi/2} \mathrm{d}\theta \\
&= \int_0^{\pi/2} \frac{\delta}{\delta - \sin\theta}\mathrm{d}\theta - \pi/2
\end{aligned}$$

Now we make a second change of variable: $t = \tan(\theta/2)$

(see http://math.stackexchange.com/questions/355897/how-to-integrate-f-theta-frac1a-sin-theta)

Then:

$$\begin{aligned}
\sin\theta &= \frac{2t}{t^2+1} \\
\mathrm{d}\theta &= \frac{2\mathrm{d}t}{t^2+1}
\end{aligned}$$

$$\theta = 0 \Leftrightarrow t = 0$$

$$\theta = \pi/2 \Leftrightarrow t = 1$$

leading to:

$$\begin{aligned}
\int_0^{\pi/2} \frac{\delta\mathrm{d}\theta}{\delta - \sin\theta} &= \int_0^1 \frac{2\delta\mathrm{d}t}{t^2+1}\frac{1}{\delta - \frac{2t}{t^2+1}} \\
&= \int_0^1 \frac{2\delta\mathrm{d}t}{\delta(t^2+1) - 2t} \\
&= \int_0^1 \frac{2\mathrm{d}t}{t^2+1-2t/\delta} \\
&= \int_0^1 \frac{2\mathrm{d}t}{(t-1/\delta)^2 + (1-1/\delta^2)}
\end{aligned}$$

With a third change of variable $u = (t - 1/\delta)/\sqrt{1 - 1/\delta^2}$:

$$\begin{aligned}
\textstyle\int_0^1 \frac{2\mathrm{d}t}{(t-1/\delta)^2+(1-1/\delta^2)} \\
&= \frac{2}{\sqrt{1-1/\delta^2}} \int_{\frac{-1}{\sqrt{\delta^2-1}}}^{\sqrt{\frac{\delta-1}{\delta+1}}} \frac{\mathrm{d}u}{u^2+1} \\
&= \frac{2}{\sqrt{1-1/\delta^2}} \left[\arctan u\right]_{\frac{-1}{\sqrt{\delta^2-1}}}^{\sqrt{\frac{\delta-1}{\delta+1}}}
\end{aligned}$$

In summary the expression of P reads:

$$\begin{aligned}
P &= \frac{KD}{l_{eq}} \\
&= \frac{KD}{r}\Big(-\frac{\pi}{4} \\
&\quad + \frac{1}{\sqrt{1-1/\delta^2}}\left[\arctan\sqrt{\frac{\delta-1}{\delta+1}} + \arctan\frac{1}{\sqrt{\delta^2-1}}\right]\Big)
\end{aligned}$$

Since $\arctan(a) + \arctan(b) = \arctan((a+b)/(1-ab))$, we simplify the expression to:

$$P = \frac{KD}{l_{eq}} = \frac{KD}{r}\left(-\frac{\pi}{4} + \frac{\delta}{\sqrt{\delta^2-1}}\arctan\sqrt{\frac{\delta+1}{\delta-1}}\right) \tag{3}$$

Eq. 3 shows that P diverges as the droplet distance decays to zero (Supplementary Fig. ??). Physically, we expect a cuttoff in the expression of P as the minimal distance between the droplet will reach a non zero thickness when droplets are pressed together. The deformation of droplets is not accounted for in our simple geometrical approximation. However, it is interesting to notice that close to the divergence, the P scales as

$$P \sim \frac{KD}{r}\frac{\pi}{2\sqrt{2}}(\delta-1)^{-1/2}, \delta \sim 1 \tag{4}$$

In contrast for larger spacing, the scaling is

$$P \sim \frac{KD}{r}\frac{1}{2}(\delta-1)^{-1}, \delta \sim \infty \tag{5}$$

The region $\delta - 1 \sim 1$ (a spacing of order the droplet size, corresponding typically to volume fractions close to 0.5) is exactly at the crossover between these two regimes. The crossover

should not be interpreted as a crossover from the diffusion limited regime to a kinetic limited regime. Here we see that simple geometrical arguments lead to this crossover in the permeability. In practice, the scaling at short distances $\delta \sim 1$ is hardly observable. For droplets of sizes of typical radius 10 μm, $\delta - 1 \sim 10^{-3}$ corresponds to spacing of about 10 nm, a scale for which a hard sphere model for the interface is not relevant. The permeability is therefore expected to plateau to values of order a few KD/r which is of order the maximal permeability we measured, obtained at the smallest spacing distance.

Supplementary Note 3: Estimation of diffusion coefficients

The translational diffusion coefficient D of a spherical particle with a radius r in liquids at low Reynolds numbers can be estimated by the Stokes-Einstein equation:

$$D = k_B T/(6\pi\eta r) \tag{6}$$

With the Boltzmann constant $k_B = 1.38\times10^{-23}\ JK^{-1}$, the dynamic viscosity of the medium η and the absolute temperature T (equal to 294 K in these experiments). The used fluorinated oil is a hydrofluorether (HFE7500, 3M). The supplier states a viscosity of η=1.24 cP. Under these conditions the diffusion coefficient of a single fluorophore molecule with a diameter of about 1 nm can be estimated to be $D = 3.5 \times 10^{-6}\mathrm{cm}^2\mathrm{s}^{-1}$. The average diameter of the surfactant assemblies measured by dynamic light scattering (Malvern Zetasizer) is about 200 nm as shown in Supplementary Fig. 3. The average diffusion coefficient of these objects is estimated at $1.4 \times 10^{-8}\mathrm{cm}^2\mathrm{s}^{-1}$ according to Eq. 6. The diffusion coefficient we measured in the minimal emulsions (of order $10^{-8}\mathrm{cm}^2\mathrm{s}^{-1}$) correlates well with the expected value for surfactant assemblies and not with the diffusion coefficient of single fluorophore molecules in the fluorinated oil: the supramolecular assemblies of surfactant therefore act as the carrier of the molecules in the transport process.

Supplementary Note 4: Water Transport

Two populations of monodisperse droplets are produced on a microfluidic chip and incubated off chip in a macroscopic vial (Skhiri, Gruner *et al.* *Soft Matter* (2012)). The volume of the droplets is measured after re-injection of the droplets into the microfluidic chip. The water transport is driven by different concentrations of sodium chloride among the droplets. To accurately determine the volume of the droplets, fluorophores are added to the droplets. As the fluorophore concentration is inversely proportional to the volume of the droplet, measuring the fluorescence signals of the droplets as a function of time allows to access the dynamics of water transport in a straight-forward maner. However, this approach is relying on the assumption that the fluorophore molecules are retained in the droplets and not released to the continuous phase, or exchanged between the droplets throughout the experiments. It has been shown that the presence of sodium chloride significantly lowers the solubility of organic molecules in an aqueous phase and hence the rate of exchange was found to be substantially enhanced, as shown in the main text. In fact, in the presence of high concentrations of sodium chloride (100 mg mL^{-1}), fluorescein sodium salt was found to be exchanged between the droplets on a relatively short timescale ($\sim$ minutes). For this reason, a fluorescently labelled dextran (Dextran, fluorescein labelled, 3000 g mol^{-1}, Invitrogen) was chosen as fluorophore encoding the size of droplets. No mass transfer of these hydrophilic polysaccharides was obtained even in the presence of high sodium chloride concentrations. Among the two droplet populations, one contains sodium chloride (100 mg mL^{-1}) and fluorescently labelled dextran (100 μM), while the other droplets consist of deionized water. For the shrinking droplet population (deionized water), the measurement of the fluorophore concentrations is not straight-forward. Below a critical volume, the droplets are not confined

between the channel walls anymore. In such a case, the fluorescence signal is not accurately measured with the presented approach. Therefore, fluorophores were only added to the population, whose droplet volume is increasing in time (containing sodium chloride). A decrease of the fluorescence signal of droplets containing sodium chloride is observed, indicating an increase of the droplet volumes. To investigate the role of the surfactant in the water transport process, the dynamics were recorded for different surfactant concentrations (0.2 wt%, 1 wt%, 5 wt%). The volume of the droplets is assumed to be inversely proportional to the measured fluorescence signal. The temporal increase of the droplet volume V normalized by the initial droplet volume V_0 is shown for different surfactant concentrations. The timescale of the water transport process is found to be independent on the surfactant concentration. Hence, the water solubility in the continuous phase mediated by the surfactant is negligible compared to the direct partitioning of water in the fluorinated oil (the solubility of water in HFE is 45 ppm by weight, value obtained from supplier 3M). This result shows that surfactant is marginally involved in the water transport process.

Supplementary Note 5: Synthesis of PFPE-PEG-PFPE Surfactant Synthesis

The synthesis protocol is adapted from Holtze *et al.* [2] and Scanga *et al.*. [3]. The molecular weight of the Krytox used is determined by ^{1}H-NMR spectroscopy using a 1:1 mixture with HFE-7500 (3M), and integrating the peak intensities of the protons of the ethoxy- and carboxilic acid groups.

The following steps are performed under inert atmosphere (Nitrogen or Argon): Krytox 157 FSH (10.0 g, 2.50 mmol (1 eq), DuPont) is dissolved in HFE7100 (30mL, 3M; dried with 4$\mathring{A}$ molecular sieves beads 8-12 mesh for one week). Oxalylchloride (2.1 mL, 25 mmol (10 eq), Sigma Aldrich, reagent grade 98%) and one drop of dimethylformamid are injected into the solution while cooling with ice. The reaction mixture is stirred until there is no further gas development. All solvents are evaporated and the product is redissolved in HFE7100 (dried, 30 mL). O,O'-Bis(2-aminopropyl) polypropylene glycol-block-polyethylene glycol-block-polypropylene glycol 500 (Jeffamine: 0.40 g, 0.38 mL, 0.67 mmol (0.27 eq) for synthesis $\mathbf{P}_1$**,** $\mathbf{P}_2$; Jeffamine: 0.85g, 0.82mL, 1.42mmol (0.57 eq) for synthesis $\mathbf{P}_3$ and Jeffamine: 0.75 g , 0.73 mL, 1.25 mmol (0.50 eq), Sigma Aldrich for synthesis $\mathbf{P}_4$**,** $\mathbf{P}_5$) is dissolved in anhydrous Tetrahydrofurane (distilled, THF, 30 ml, Sigma Aldrich, $\geq$ 99.9%) and Triethylamine (1.73 mL, 12.5 mmol (5 eq), Sigma Aldrich, $\geq$ 99%) is added to the solution. $\mathbf{P}_0$ is synthesized using a similar scheme although without the drop of dimethylformamid (E. Mayot, private communication). The HFE7100 solution is then injected into the THF solution while stirring. The solution is stirred overnight. At the end, the product is stable to be handled under atmospheric conditions.

We performed a control synthesis according to the previous protocol, where the reaction

mixture is briefly opened to air after the Oxalylchloride has reacted with the Krytox (C_1), showing the crucial role of the inert atmosphere on the synthesis.

All solvents are evaporated and the product is redissolved in FC3283 (30mL, 3M), filtrated and washed (3x25mL) over Celite 545 (Sigma Aldrich) using a glass frit (porosity 2) before being dried under vacuum. Filtering twice reduces the yield and does not improve the purity. The final milky semisolid product is obtained with yields of 50-90%, the loss being mainly related to filtration.

Supplementary Note 6: Analysis of PFPE-PEG-PFPE Surfactant Synthesis

The analytical protocol is based on Infra-Red (IR) spectroscopy [4, 5] according to Ghenciu *et al.* [6], Dejournette *et al.* [1] and Matochko *et al.* [7].

From the reaction scheme, we expect two side products, as described previously, the carboxylic acid and the carboxylate salt which might arise from a triethylamine salt or from a Jeffamine salt[1]. The product should display both an amide bond and the characteristic Jeffamine bonds.

The IR spectra were normalised to the band of the alkane group of the Jeffamine between 3000 cm^{-1} and 2800 cm^{-1} (R_2). Small variations exist between the batches of Jeffamine, the spectrum P_0 corresponds to the surfactant used in this study (Supplementary Fig. 5(A)). The Krytox does not show strong bands in the range of 3000 cm^{-1} to 2800 cm^{-1}, but the carboxylate groups give peaks in the range of 1600 - 1800 cm^{-1} where the Jeffamine does not give any band. The Krytox (R_0) shows a clear band at 1775 cm^{-1}, as previously reported by Doan *et al.* [8] and Loeker *et al.* [9]. For all products (P_0 to P_5), we measure a band related to the amine bands which is systematically shifted (compared to the Krytox band) indicating that the reaction occured (Supplementary Fig. 5(B)).

For the control reaction (reaction opened to air, C_1), we cannot find any band in the range of 3000-2800 cm^{-1}: the jeffamine is not covalently bound to the Krytox, the reaction did not occur. The product is however different than the reagent (product band at 1700 cm^{-1}). In the literature, a shift of the band towards lower wavenumbers is attributed to a carboxylate-ammonium salt (Figure 2, turquoise curve in [1]). The 1700 cm^{-1} band is also present in Figure 1 of Matochko et al [7]. As a note, it is expected that the CO band of the salt shifts

with the counterion: we observed for example a band at 1650 cm^{-1} for the ammonium salt of Krytox (see control C_2), as reported by Loeker *et al.* previously [9] while sodium salts lead to bands (eventually doublets) at 1665 - 1696 cm^{-1} [8]. As the alkane band of the Jeffamine is not present (in control C_1), we conclude, that the band at 1700cm^{-1} corresponds to a salt of Krytox (likely with a triethylammonium counterion used in the synthesis for the control C_1).

Considering now the IR spectra of our products P_{0-5}, we systematically obtain a clear band at 1720 cm^{-1}. In Dejournette *et al.* [1]), this band is recovered for the covalently bound Krytox-jeffa (Figure 2 in [1], black curve) but with a strong additional band at 1770 cm^{-1} where we found the reagent Krytox band. The product of the reaction in the Dejournette paper is therefore highly contaminated with Krytox. The 1720 cm^{-1} band is also present in Matochko *et al.* [7] but is a shoulder of the peak at 1700 cm^{-1} (Figure 1 (B,C) in [7]): the product of the reaction in Matochko *et al.* [7] is therefore likely to be contaminated with the Krytox salt.

In contrast, all of our products show minor bands at 1775 cm^{-1} and – possibly – a very weak shoulder around 1700 cm^{-1}. Therefore the purity of our surfactant is much higher than that of previous publications. We also recover that when the reaction is performed with an excess of Krytox (P_1, P_2), the side product is mainly the Krytox (bump at 1780-1790 cm^{-1}). In contrast, when the reaction is performed with larger amounts of Jeffamine (P_3,P_4,P_5), the side product is mainly the jeffamine-Krytox salt.

In our synthesis, the amount of Krytox left over (in the form of acid or salt) is small, in the order of a couple of percent from an estimate based on the spectra and better than previous claims [2]. In order to estimate this amount quantitatively, we use the partitioning experiment with Rhodamine 6G (see Figure 7(E,F) in the main text). We have shown

that Krytox binds to organic molecules as a 1:1 complex leading to extraction towards the oil. For the samples P_{0-5}, we used a 0.5% PEG-PFPE surfactant solution and measured the fluorescence in the oil phase (Supplementary Figure 5(C). We compare the amount of extracted Rhodamine 6G to the amount with extraction with known amount of carboxylic acid. All surfactant solutions show a partitioning to the oil corresponding to less than 1% contamination with Krytox. The purity of our end product is therefore of order 99%.

SUPPLEMENTARY REFERENCES

[1] DeJournette, C. J. *et al.* Creating biocompatible oil-water interfaces without synthesis: direct interactions between primary amines and carboxylated perfluorocarbon surfactants. *Anal Chem* **85**, 10556–10564 (2013). URL `http://dx.doi.org/10.1021/ac4026048`.

[2] Holtze, C. *et al.* Biocompatible surfactants for water-in-fluorocarbon emulsions. *Lab Chip* **8**, 1632–1639 (2008). URL `http://dx.doi.org/10.1039/b806706f`.

[3] Scanga, R. *et al.* Fluorinated amphiphilic block copolymers to stabilize water-in-fluorocarbon emulsions. *Polymer Preprint* **50**, 148 (2009).

[4] Lide, D. R. (ed.) *CRC Handbook of Chemistry and Physics* (CRC Press, Boca Raton, FL, 2005).

[5] Nakanishi, K. . *Infrared absorption spectroscopy, practical.* (San Francisco: Holden-Day., 1962).

[6] Ghenciu, E. & Beckman, E. Affinity extraction into carbon dioxide. 1. extraction of avidin using a biotin-functional fluoroether surfactant. *Ind. Eng. Chem. Res.* **36**, 5366–5370 (1997).

[7] Matochko, W. L. *et al.* Uniform amplification of phage display libraries in monodisperse emulsions. *Methods* **58**, 18–27 (2012). URL `http://dx.doi.org/10.1016/j.ymeth.2012.07.012`.

[8] Doan, V., Koeppe, R. & Kasai, P. Dimerization of carboxylic acids and salts: An ir study in perfluoropolyether media. *JACS* **119**, 9810–9815 (1997).

[9] Loeker, F., Marr, P. & Howdle, S. Ftir analysis of water in supercritical carbon dioxide microemulsions using monofunctional perfluoropolyether surfactants. *Colloids and Surfaces A: Physicochemical and Engineering Aspects* **214**, 143–150 (2003).
